# Habitat selection of an endangered primate, the samango monkey (*Cercopithecus albogularis schwarzi*): Integrating scales to prioritize habitat for wildlife management

**DOI:** 10.1002/ece3.7631

**Published:** 2021-05-05

**Authors:** Edwin J. Parker, Nicola F. Koyama, Russell A. Hill

**Affiliations:** ^1^ School of Biological & Environmental Sciences Liverpool John Moores University Liverpool UK; ^2^ Primate and Predator Project Lajuma Research Centre Louis Trichardt South Africa; ^3^ Department of Anthropology Durham University Durham UK; ^4^ Department of Zoology University of Venda Thohoyandou South Africa

**Keywords:** connectivity, conservation, habitat fragmentation, indigenous forest, resource selection function, samango monkey

## Abstract

**Aim:**

As habitat loss continues to accelerate with global human population growth, identifying landscape characteristics that influence species occurrence is a key conservation priority in order to prevent global biodiversity loss. In South Africa, the arboreal samango monkey (*Cercopithecus albogularis* sp.) is threatened due to loss and fragmentation of the indigenous forests it inhabits. The aim of this study was to determine the habitat preferences of the samango monkey at different spatial scales, and to identify key conservation areas to inform management plans for this species.

**Location:**

This study was carried out in the western Soutpansberg Mountains, which represents the northernmost population of samango monkeys within South Africa, and the only endangered subspecies (*C. a*. *schwarzi*).

**Methods:**

We used sequentially collected GPS points from two samango monkey groups followed between 2012 and 2017 to quantify the used and available habitat for this species within the western Soutpansberg Mountains. We developed 2nd‐order (selection of ranging area), 3rd‐order (selection within range), and 4th‐order (feeding site selection) resource selection functions (RSFs) to identify important habitat features at each scale. Through scale integration, we identified three key conservation areas for samango monkeys across Limpopo Province, South Africa.

**Results:**

Habitat productivity was the most important landscape variable predicting probability of use at each order of selection, indicating the dependence of these arboreal primates on tall‐canopy indigenous forests. Critical habitat across Limpopo was highly fragmented, meaning complete isolation between subpopulations is likely.

**Main conclusions:**

Understanding the habitat characteristics that influence samango monkey distribution across South Africa is crucial for prioritizing critical habitat for this species. Our results indicated that large, contiguous patches of tall‐canopy indigenous forest are fundamental to samango monkey persistence. As such, protected area expansion of large forest patches and creation of forest corridors are identified as key conservation interventions for this species.

## Graphical abstract

Identifying the landscape characteristics that influence species occurrence is a key conservation priority in order to prevent global biodiversity loss. We used samango monkey GPS locations over a 5‐year study period to determine the habitat characteristics most important to their distribution at multiple scales. We found that habitat productivity was the most important landscape variable predicting probability of use at each scale of selection, and delineate the probability of samango monkey use across their known range in Limpopo Province, South Africa, accordingly.

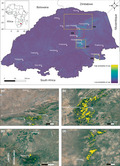

## 1 INTRODUCTION

With the accelerated conversion of land for agriculture and urbanization coinciding with the rising global human population, loss and fragmentation of natural habitat are occurring at an unprecedented rate (Estrada et al., 2017; Haddad et al., 2015). The destruction and degradation of natural habitat are the primary causes of declining global biodiversity (Fahrig, 2003; Lindenmayer & Fischer, 2006). Natural habitat loss leads to fragmentation, creating smaller and more isolated patches of suitable habitat separated by a matrix of unsuitable or human‐modified habitat (Fahrig, 2003; Haddad et al., 2015). Fragmentation can restrict populations to discrete patches of suitable habitat (Fahrig, 2003), reducing connectivity within the landscape and therefore impacting metapopulation dynamics (Dolrenry et al., 2014; Ricketts, 2001). Connectivity increases gene flow between populations (Stockwell et al., 2003), which ultimately facilitates species persistence (Doerr et al., 2011) and mitigates the effects of habitat disturbance (Olds et al., 2012).

In light of accelerated habitat loss and fragmentation, understanding the factors that influence species occurrence and persistence is fundamental to the development of effective management plans and preventing biodiversity loss (Kopp et al., 1998; Mayor et al., 2009). The relative importance of a resource to a species varies with the scale of interest (Boyce, 2006), and thus, conservation/management planning is highly scale‐dependent (Mayor et al., 2009). Resource selection functions (RSFs) (Manly et al., 2002) are an invaluable tool for identifying important resources required by a species at multiple scales (Boyce, 2006; DeCesare et al., 2012). RSFs are statistical models that estimate the relative probability of use of a particular resource unit by an individual or group (Manly et al., 2002) and can be used to map distribution and connectivity across the landscape.

Due to the accessibility of radio telemetry and remotely sensed environmental data, the majority of RSF studies have focused on wide‐ranging species, particularly large carnivores (Davidson et al., 2012; Dellinger et al., 2013; Fattebert et al., 2015; Pitman et al., 2017; Simcharoen et al., 2008) and herbivores (DeCesare et al., 2012; Mancinelli et al., 2015; Roever et al., 2012; van Beest et al., 2010). Despite this, RSFs may be particularly valuable in conservation planning for small‐ranging and patchily distributed species living in fragmented habitats, by identifying critical habitat both within and between fragments (Harris et al., 2008). Despite the imperiled conservation status of many of the world's primates (Estrada et al., 2017) and the resolution of data gained through habituation and observational methods, studies using RSFs to model resource selection in primates are almost entirely lacking (Sawyer & Brashares, 2013).

The samango monkey (*Cercopithecus albogularis* subspp., Dalton et al., 2015) represents Africa's most southerly arboreal guenon. Its distribution throughout South Africa is closely associated with the prevalence of indigenous forest types such as Afromontane/Mistbelt, Scarp, and Coastal belt forests (Hayward et al., 2005; Lawes, 1990), which they heavily rely on for various aspects of their ecology, including food and protection (Coleman & Hill, 2014b; Nowak et al., 2017; Parker et al., 2020; Wimberger et al., 2017). As with other arboreal primates, samango monkeys are highly susceptible to fragmentation and human‐induced landscape change due to their dependence on tall, closed‐canopy forests (Chapman et al., 2000). In southern Africa, natural habitats are decreasing due to anthropogenic conversion of land for agriculture and urbanization (Friedmann & Daly, 2004; Kingdon et al., 2012), meaning samango monkeys must exist in isolated or semi‐isolated forest fragments with little or no connectivity between patches (Dalton et al., 2015; Lawes et al., 2000; Linden et al., 2016; Swart & Lawes, 1996). This is further compounded by the poor dispersal capabilities of samango monkeys and their reluctance to traverse open ground (Lawes, 1992, 2002; Lawes et al., 2000).

The samango monkeys living in the Soutpansberg Mountains represent an isolated population of the most vulnerable of the three samango monkey subspecies in South Africa (*C. a*. *schwarzi*; Dalton et al., 2015) and are currently listed as endangered on the national Red List (Linden et al., 2016). Key conservation interventions for this species identified in the Red List assessment include protected area expansion of large forest patches and the creation and maintenance of forest corridors connecting forest patches (Lawes et al., 2000; Linden et al., 2016; Swart & Lawes, 1996). Given the high conservation value of maintaining and restoring areas of suitable habitat and connectivity (Crooks & Sanjayan, 2006), identifying priority areas and areas of potential connectivity is critically important for the long‐term persistence of this species.

Resource selection studies can be used to prioritize suitable habitat and develop effective management plans. Here, we estimate samango monkey resource selection within the Soutpansberg Mountains, South Africa, at multiple spatial scales according to Johnson (1980): 2nd‐order selection (selection within geographic range), 3rd‐order selection (selection within home range), and 4th‐order selection (selection of feeding sites). To aid management efforts, we then identify critical habitat to infer both persistence within and connectivity between subpopulations across the samango monkey range in Limpopo Province (hereafter “Limpopo”), South Africa, using scale integration (DeCesare et al., 2012; Johnson et al., 2004).

As forest specialists, samango monkeys heavily rely on patches of tall‐canopy indigenous forest for food and protection (Linden et al., 2016; Nowak et al., 2017; Parker et al., 2020; Wimberger et al., 2017), and avoid large open areas (Lawes, 1992, 2002; Lawes et al., 2000). We therefore predicted that samango monkeys would strongly select for areas of high primary productivity at all levels of selection. We also predicted that samango monkeys would avoid areas of high terrain ruggedness within the home range and when selecting feeding sites, as these areas are associated with open, barren cliffs across the mountain range, but that these areas would be selected when establishing home ranges due to the location of indigenous forests on the south‐facing cliffs (Mostert et al., 2008). Given the location of the study groups on the mountain range and the association of certain habitat types at specific elevations (Mostert et al., 2008), we predicted that samangos would select for areas of high elevation when establishing home ranges, but that this preference would be less pronounced within the home range and when selecting feeding sites. As samango monkeys are capable of utilizing riverine forests for dispersal between indigenous forest patches, we predicted that monkeys would select areas close to rivers at each order of selection (Lawes, 1992; Linden et al., 2016). Finally, due to the risk associated with human settlements (Nowak et al., 2017; Wimberger et al., 2017), samango monkeys should avoid areas close to human settlements across all orders of selection.

## 2 METHODS

### 2.1 Study area and data collection

Our study was conducted within the Soutpansberg Mountains, Limpopo, South Africa (29°26′05″E, 23°02′23″S), part of the 6,800 km^2^ UNESCO Vhembe Biosphere Reserve. The Soutpansberg Mountains represent the northernmost distribution of samango monkeys within South Africa (Dalton et al., 2015) and the northernmost population of the subspecies *C. a*. *schwarzi* within Limpopo (Dalton et al., 2015). The mountain range experiences large seasonal variation in rainfall and temperature, in addition to substantial variation in elevation and water availability, resulting in a variety of vegetation types (Mostert et al., 2008). On the north‐facing slopes, montane grasslands, open woodlands, and leached sandveld dominate due to the arid conditions and high elevation (Mostert et al., 2008). In contrast, indigenous evergreen forests (described as northern mistbelt; Mostert et al., 2008; Mucina & Rutherford, 2006) dominate the south‐facing ridges as a result of direct mist precipitation and the collection of groundwater from the base of the cliffs. Further downslope of the mistbelt forest, semi‐deciduous woodland, and thicket forest become more abundant, while riverine forests occur along the streams heading down the mountains (Hahn, 2006). These vegetation types are further fragmented by farmland and commercial plantations, while urban settlements become more prevalent at the base of the mountains (Mostert et al., 2008).

### 2.2 Location data

All behavioral data collection followed the Association for the Study of Animal Behaviour (ASAB) Guidelines for the Treatment of Animals in Behavioural Research and Teaching (ASAB, 2012) and complied with the University's use of Live Animals in Unregulated Research guidelines (NK_EP/2016‐10). All fieldwork was approved by the Animal Welfare Ethical Review Board and the Department of Anthropology Ethics Committee at Durham University, UK, and was conducted with approved permits from Limpopo Province Department of Economic Development, Environment and Tourism (LEDET).

Data were collected on two habituated groups of samango monkeys (*C. a*. *schwarzi*) at the Primate and Predator Project, Lajuma Research Centre, in the western Soutpansberg Mountains. Samango monkeys are arboreal, diurnal guenons, which form single‐male, multifemale groups (Henzi & Lawes, 1987) normally of around 30 individuals (Coleman & Hill, 2014a; Lawes et al., 2013). Home range size typically varies between 0.15 and 0.46 km^2^ depending on subspecies (Linden et al., 2016; Parker et al., 2020). However, group sizes at Lajuma were 30–40 individuals (“Barn” group) and 60–70 individuals (“House” group), with average home range sizes of 0.56 km^2^ (±0.07) and 0.60 km^2^ (±0.13), respectively (Parker et al., 2020). Each group was followed for an average of 8 full days every month between 2012 and 2017, with full days defined as those where a group was followed from morning sleep site to evening sleep site without losing audiovisual contact for more than 60 min. GPS points of the groups' location (with a location error of ±5 m) were taken from a central position within each group, using a handheld GPS device (Garmin GPSMAP 64S), every 20 min to coincide with scan samples collecting behavioral data on feeding (feeding/foraging) (Altmann, 1974).

As spatial data are inherently autocorrelated (Gillies et al., 2006), we thinned our “used” sample at each order of selection (Northrup et al., 2013) to one GPS fix every 4 hr between 6 a.m. and 6 p.m., resulting in four GPS fixes per day. We defined samango monkey “used” locations for 2nd‐order (selection of ranging area) and 3rd‐order (selection within home range) analysis as all four‐hourly GPS fixes for each group between 2012 and 2017, within each group's 95% volume isopleths derived from adaptive localized convex hulls (a‐LoCoH) (Getz et al., 2007; Getz & Wilmers, 2004). This resulted in 2,470 locations for Barn group and 2,288 locations for House group. Finally, we defined “used” locations for 4th‐order analysis (selection of feeding areas) as all GPS fixes from four‐hourly scan samples where feeding occurred in over 50% of the total number of scanned individuals per 5‐min sample (using a minimum scan sample size of six individuals), within each group's 95% isopleth. This resulted in 908 and 942 “feeding” locations for Barn and House group, respectively.

### 2.3 Resource selection function training

To model habitat selection at multiple scales, we built RSFs (Manly et al., 2002) in a used–available design at the 2nd‐, 3rd‐, and 4th‐order scales of selection (Johnson, 1980). We defined the area available to samango monkeys for selection of ranging area (2nd‐order selection) as the western range of the Soutpansberg Mountains, due to the occurrence of samango monkey groups across this extent and thus theoretically depicting the area available to samango monkeys. For selection within range (3rd order), we considered the area available to be the annual minimum convex polygon (MCP) for each group, respectively. Finally, we considered the area within a 1.5‐km buffer of each feeding location to be the area available for feeding site selection (4th order), which is the average daily path length of samango monkeys recorded at the study site (Parker et al., 2020) and thus the area theoretically available when selecting feeding sites.

To sample available locations, we generated random points using the “Random points inside polygon” function within the “Research Tools” toolbox in QGIS (v2.18, QGIS Development Team, 2017) within the available area designated at each order of selection. We created available locations at a 1:10 ratio of used to available locations (Koper & Manseau, 2012). Available locations were created at this ratio to create a sufficiently large available sample in order to accurately approximate the point process model and allow correct inference from model coefficients (Northrup et al., 2013). While the used–available design of RSFs means that some of the available locations may have in fact been used (known as pseudoabsences), deterministic selection of the available sample allows RSFs to control for this by best approximating the point process likelihood (Johnson et al., 2013). We sampled landscape variables at each used and available location using the “Point sampling tool” plugin in QGIS. Landscape variables sampled were annual EVI (Enhanced Vegetation Index—a remotely sensed measure of productivity) for each year across the study period, terrain ruggedness, elevation, distance to rivers, and distance to human settlements. We opted to use EVI in our analysis over more conventional land cover classes due to the greater resolution afforded by EVI composites and the increased sensitivity and responsiveness to canopy structure and composition (Pettorelli et al., 2005), factors that are likely to be important for an arboreal species (Parker et al., 2020).

We obtained annual EVI layers across the western Soutpansberg Mountains from Landsat 8 datasets from Google Earth Engine (earthengine.google.com) at a 30 m^2^ resolution. Annual EVI represents the average productivity of a given cell across a year. We used annual EVI due to the scale of analysis, and for comparability with other studies (Sawyer & Brashares, 2013). We obtained the elevation layer from NASA's Shuttle Radar Topography Mission dataset, also downloaded from Google Earth Engine at a 30 m^2^ resolution across the study area, and calculated terrain ruggedness from this layer using the “Terrain Analysis” toolbox in QGIS. In order to interpolate resource selection across Limpopo, we also sampled these three variables across Limpopo at a coarser resolution (250 m^2^), which was the finest resolution available at this much larger spatial scale due to gaps in the data at a 30 m^2^ resolution. A “Distance to rivers” raster layer was created across Limpopo by downloading a rivers layer from the South African Department of Water and Sanitation website (dwa.gov.za) and converting this to a “distance to” layer using the “Proximity” function in the “Analysis” toolbox in QGIS. Finally, a “Distance to human settlements” raster layer was created across Limpopo by extracting urban and agricultural areas from the South African National Land Cover dataset (2018) (land cover classes 47–67), using QGIS's “Raster Calculator,” and converting this to a “distance to” layer using the “Proximity” function in the “Analysis” toolbox. We used the latest national land cover dataset for this analysis (Thompson, 2018) due to the updated location of areas of human activity, particularly areas in close proximity to the study groups, which would have biased model coefficients, leading to inaccurate predictions when mapping probability of use across the landscape. We also included major roads in our “Distance to human settlements” variable as roads represent a major barrier to samango monkey dispersal and cause numerous fatalities to samango monkeys across the mountain range (Linden et al., 2020). Roads could not be included as a separate variable due to collinearity with urban areas.

We designed generalized linear mixed‐effects models (GLMMs) each with a binomial error structure (1 = used and 0 = available) and a logit link function to model annual samango monkey habitat selection at multiple scales. Datasets were randomly subset into 80% training and 20% testing datasets to allow external model validation using cross‐validation, a method shown to be the most appropriate for used–availability RSF models (Johnson et al., 2006). Analysis was carried out at the annual level for comparability with other studies (Sawyer & Brashares, 2013). We included group (“Barn/House”) as a fixed effect as this variable only had two levels and could not be included as a random effect (Bolker et al., 2008). Year was included as a random variable to control for potential differences in selection between years. All landscape variables sampled were included in the analysis creating a maximal model (Hurvich & Tsai, 1990). Models were fitted in R 3.4 (R Core Team, 2017) using the glmer function in the “lme4” package (Bates et al., 2014). Model coefficients were standardized prior to model fitting to allow for comparison between scales of selection.

We assessed model stability by comparing the estimates of a model based on all the data with those obtained from models excluding levels of the random effects one at a time using the influence function within the “influence.ME” package in R (Nieuwenhuis et al., 2012), which indicated that the models were stable. Fixed effects were explored for collinearity using variance inflation factors derived from a standard generalized linear model excluding the random effects, using the vif function within the “car” package with a cutoff value of 2 (Hair et al., 2014). Variables above this threshold were removed from the resulting models to create more parsimonious models. Significance for *p*‐values of the individual effects was inferred at the 5% level. Confidence intervals were estimated using the confint function in the “lme4” package.

### 2.4 RSF validation

We projected the predicted relative probability of samango monkey use at each selection level across the study area in QGIS following Manly et al. (2002). We then reclassified raw RSF values into 10 equally sized bins (Boyce et al., 2002) and counted the frequency of the withheld used locations that fell into each bin. We used a Spearman rank correlation to test the frequencies of used locations observed in each RSF bin (Johnson et al., 2006), with a significant positive correlation between RSF bin rank and frequency of used points indicative of the predictive ability of the RSF model (Boyce et al., 2002; Johnson et al., 2006).

### 2.5 RSF projection and scale integration

The used–available designs at each scale (2nd‐, 3rd‐, and 4th‐order) generate RSFs that are proportional to the probability of use (DeCesare et al., 2012; Johnson et al., 2006; Manly et al., 2002). We spatially mapped probability of use at each order across the study area by estimating predicted RSF values per 30 m^2^ pixel according to Manly et al. (2002). Predicted RSF values were scaled between 0 and 1 using a linear stretch (Johnson et al., 2004). We combined the predicted RSF values for each spatial scale to develop a scale‐integrated RSF (SRSF) (DeCesare et al., 2012; Johnson et al., 2004; Pitman et al., 2017) at a resolution of 250 m^2^ across Limpopo and applied a linear stretch to scale RSF values between 0 and 1. To delineate critical habitat and key conservation areas for *C. a*. *schwarzi* across their known range within Limpopo (Linden et al., 2016), we spatially mapped habitat where probability of use was >2/3 (Heinrichs et al., 2010).

## 3 RESULTS

Cross‐validation of our RSFs with the withheld data revealed a strong positive correlation between RSF bin rank and number of observed points (Spearman's rank correlation: 2nd‐order selection: *r*_s_, 0.88, *p* < 0.001; 3rd‐order selection: *r*_s_, 0.74, *p* = 0.01; 4th‐order selection: *r*_s_, 0.85, *p* = 0.002), thus demonstrating the strong predictive capabilities of our models in delineating probability of samango monkey use across the Soutpansberg Mountains.

### 3.1 **Selection of ranging area (2**nd**‐order selection)**

**FIGURE 1:**
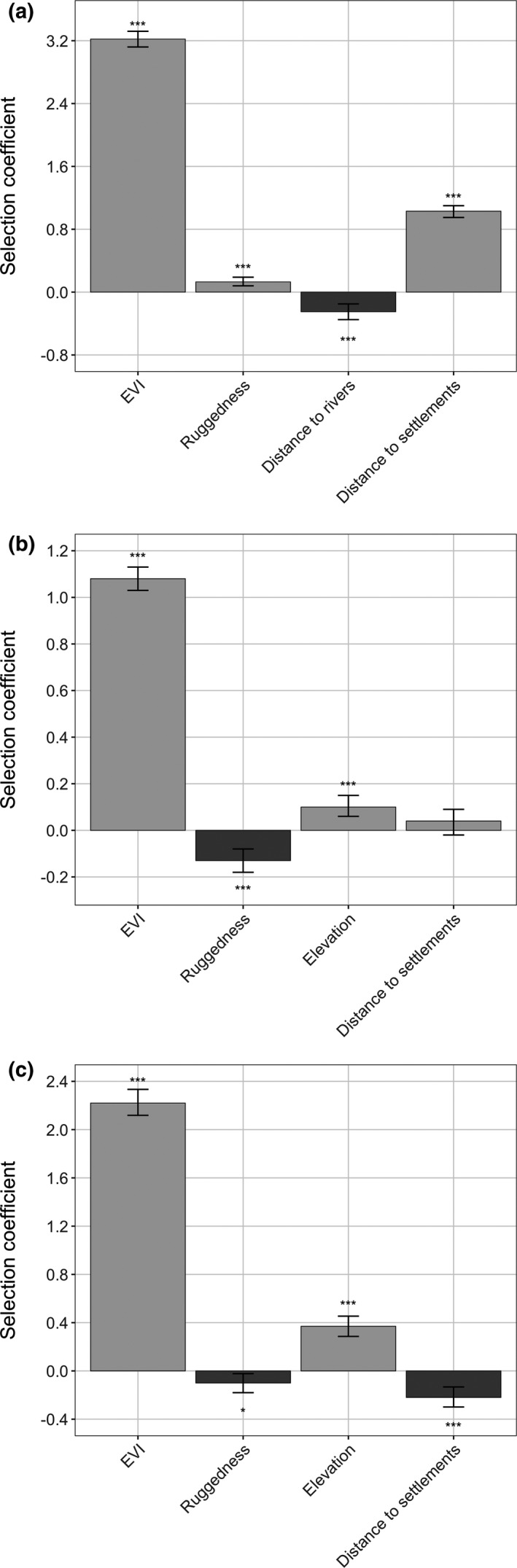
Selection coefficient estimates and 95% confidence intervals of landscape variables for 2nd‐order (selection of ranging area; a), 3rd‐order (selection within range; b), and 4th‐order (feeding site selection; c) for samango monkeys (*C. a*. *schwarzi*) in the Soutpansberg Mountains, South Africa. *Significance at 5%

Elevation was removed from our 2nd‐order RSF due to collinearity with other variables. Habitat productivity (indicated by EVI) was the most important landscape variable for samango monkeys when establishing home ranges (Figure 1a, Table 1), with a clear preference for areas of high EVI indicative of tall‐canopy, dense forests. Samango monkeys strongly avoided areas close to human settlements when establishing home ranges, while also showing a preference toward areas closer to main rivers and areas of greater terrain ruggedness.

**TABLE 1:** Coefficient estimates and key statistics for 2nd‐order (selection of ranging area), 3rd‐order (selection within range), and 4th‐order (feeding site selection) resource selection functions of samango monkeys (*C. a*. *schwarzi*) in the Soutpansberg Mountains, South Africa, 2012–2017

Coefficient	*ß*	*SE*	CI_lower_	CI_higher_	*p*
2nd order					
Intercept	−5.83	0.45	−6.87	−4.79	(1)
EVI	3.22	0.05	3.12	3.32	<0.001
Ruggedness	0.13	0.03	0.08	0.19	<0.001
Distance to rivers	−0.25	0.05	−0.35	−0.15	<0.001
Distance to settlements	1.03	0.04	0.95	1.10	<0.001
Group (house)	−0.03	0.05	−0.14	0.08	0.57
3rd order					
Intercept	−2.69	0.15	−3.04	−2.34	(1)
EVI	1.08	0.03	1.03	1.13	<0.001
Ruggedness	−0.13	0.02	−0.18	−0.08	<0.001
Elevation	0.10	0.02	0.06	0.15	<0.001
Distance to settlements	0.04	0.03	−0.02	0.09	0.18
Group (house)	0.18	0.05	0.09	0.27	<0.001
4th order					
Intercept	−3.45	0.31	−4.20	−2.71	(1)
EVI	2.22	0.05	2.12	2.33	<0.001
Ruggedness	−0.10	0.04	−0.18	−0.02	0.01
Elevation	0.37	0.04	0.29	0.45	<0.001
Distance to settlements	−0.22	0.04	−0.30	−0.13	<0.001
Group (house)	0.02	0.06	−0.10	0.15	0.70

Note

EVI, enhanced vegetation index; Ruggedness, terrain ruggedness; Distance to settlements, distance to human settlements (agricultural and urban areas). (1) Not shown because of having no meaningful interpretation.

### 3.2 **Selection within home range (3**rd**‐order selection)**

Distance to rivers was removed from our 3rd‐order RSF due to collinearity with other variables. Selection within the home range again favored areas of higher productivity, which was the landscape variable most strongly selected for (Figure 1b, Table 1). Samango monkeys also disproportionately used areas of lower terrain ruggedness and higher elevation, while distance from human settlements did not influence selection within ranges.

### 3.3 **Feeding site selection (4**th‐**order selection)**

Distance to rivers was removed from our 4th‐order RSF due to collinearity with other variables. Feeding site selection followed a similar pattern to the other orders of selection, in that highly productive areas were the landscape variable most strongly selected for (Figure 1c, Table 1). Low terrain ruggedness and high elevations were also important when selecting feeding sites. In contrast to the other orders of selection, samango monkeys used areas closer to human settlements when feeding.

### 3.4 RSF projection

**FIGURE 2:**
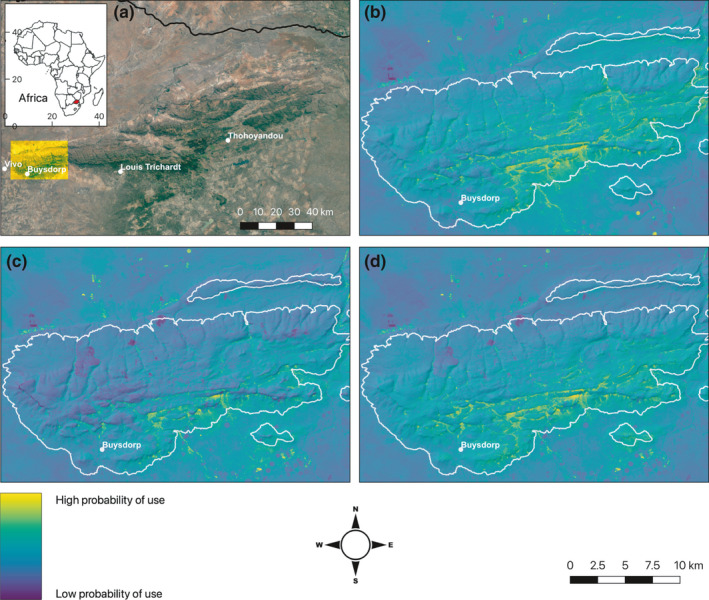
Location of study area within the Soutpansberg Mountains, South Africa, (yellow overlay, a) and location of Soutpansberg Mountains within Africa (inset, red, a), probability of use with respect to 2nd‐order (selection of ranging area; b), 3rd‐order (selection within range; c), and 4th‐order (selection of feeding site; d) by samango monkeys. Outline of mountain range above 1,000 m highlighted in white

Projection of 2nd‐, 3rd‐, and 4th‐order RSFs across the western Soutpansberg Mountains (Figure 2) showed that the highly productive plateaus on the south‐facing side of mountain consistently had the highest probability of use. These areas were predominantly associated with the tall‐canopy, evergreen indigenous mistbelt forest and, to a lesser extent, woodland and thicket (Mostert et al., 2008; Parker et al., 2020). The apparent suitability of some fields and farms off the mountain was an artifact of their high EVI resulting from fertilization and pivot irrigation.

**FIGURE 3:**
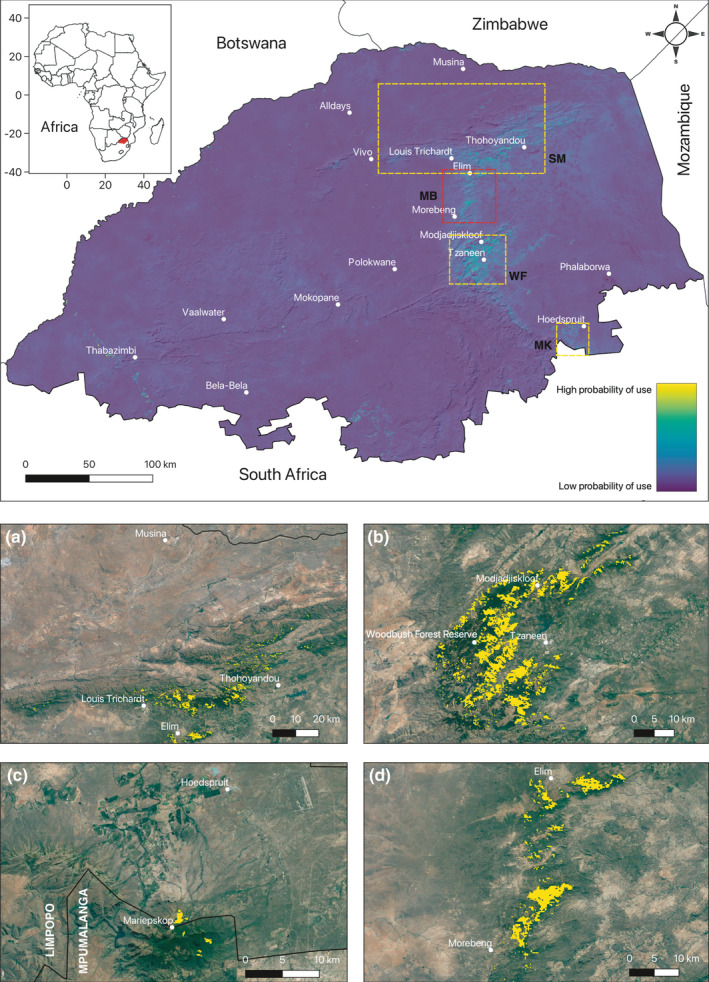
Scale‐integrated resource selection function (SRSF) predicting probability of use by samango monkeys across Limpopo Province, South Africa (top). Three key conservation areas are identified (yellow dashed box) based on existing samango monkey records: Soutpansberg Mountains (SM, a), Woodbush Forest Reserve (WF, b), and Mariepskop (MK, c), with a potential fourth key area northeast of Morebeng (red dashed box) also shown (MB, d). Inset: location of Limpopo (red) within Africa. Populated cities, towns, and suburbs (white, lower case) and province boundaries (white, upper case) are also shown to indicate locality

Projection of the scale‐integrated RSF (SRSF) across Limpopo identified three key conservation areas for samango monkeys: the Soutpansberg Mountains (Figure 3a), the Woodbush Forest Reserve (Figure 3b), and Mariepskop (Figure 3c). We also highlight a potential fourth key conservation area (Figure 3d) located between the Soutpansberg Mountains and Woodbush Forest Reserve populations. Suitable habitat across Limpopo was highly fragmented with little connectivity between populations.

The total area of critical habitat across Limpopo was 431.2 km^2^. The Woodbush Forest Reserve, with an area >220 km^2^, comprised the largest area of critical habitat of the three key samango monkey conservation areas (Table 2) and was also the area with the greatest mean patch area 0.58 km^2^. The Soutpansberg Mountains was the second largest area (98.1 km^2^), with Mariepskop representing the smallest area of critical samango monkey habitat within Limpopo (2.4 km^2^). The potential area of critical habitat northeast of Morebeng comprised 66.9 km^2^. However, the Soutpansberg Mountains had the highest number of patches of all the key areas, suggesting patches of suitable habitat were highly fragmented across this range.

**TABLE 2:** Area statistics for key samango monkey conservation areas identified from the integrated RSF across Limpopo Province, South Africa

Population	Critical habitat (km^2^)	Number of patches	Largest patch (km^2^)	Smallest patch (km^2^)	Mean patch area (km^2^)
Soutpansberg	98.1	668	6.4	<0.001	0.15
Morebeng	66.9	122	23.4	<0.001	0.55
Woodbush	229.1	398	47.3	<0.001	0.58
Mariepskop	2.4	7	1.4	0.01	0.34
Total	431.2	2,578	47.3	<0.001	0.17

## 4 DISCUSSION

Our study investigated habitat selection at the 2nd‐, 3rd‐, and 4th‐order (Johnson, 1980) of the endangered samango monkey in the Soutpansberg Mountains, South Africa. We found that selection for areas of high productivity, associated with the indigenous mistbelt forest, was the landscape variable most strongly selected for across all three orders of selection. In contrast, selection of other landscape variables differed with the scale of interest. By integrating selection across each scale and projecting the probability of use across Limpopo Province, South Africa, we were able to identify three key conservation areas based on habitat suitability and documented samango monkey presence. We also highlight a potential fourth key conservation area based on habitat suitability but where samango monkey presence, to our knowledge, has not currently been confirmed. However, our scale‐integrated RSF (SRSF) indicated substantial fragmentation between samango monkey populations with little or no connectivity that highlights a significant conservation challenge.

We found that habitat productivity (indicated by remotely sensed EVI) was the most important landscape variable influencing samango monkey habitat use across all scales of selection. High EVI values are indicative of tall‐canopy, dense vegetation (Huete et al., 2002, 2006) and are associated with the evergreen northern mistbelt forest across Limpopo (Mostert et al., 2008; Mucina & Rutherford, 2006; Parker et al., 2020). A strong selection toward areas of dense, tall‐canopy forests is unsurprising for an arboreal species (Liu et al., 2019; Palminteri & Peres, 2012; Vidal‐Garcia & Serio‐Silva, 2011) and is consistent with other studies demonstrating the dependence of samango monkeys on areas of mistbelt forest (Coleman & Hill, 2014b; Linden et al., 2016; Nowak et al., 2017; Parker et al., 2020; Wimberger et al., 2017). These forests contain important fruit species for samango monkeys (Linden et al., 2015; Nowak et al., 2017; Wimberger et al., 2017), while also affording protection from predators (Coleman & Hill, 2014b; Nowak et al., 2014, 2017; Parker et al., in review). In addition, sleeping trees are also disproportionately found within the mistbelt forest (Coleman, 2013). While samangos can utilize matrix and nonforested habitat while foraging, dispersing, and moving between forest patches (Emerson & Brown, 2013; Heikamp, 2008; Lawes & Piper, 1992; Wimberger et al., 2017), they are ultimately restricted by access to indigenous forest patches (Nowak et al., 2017; Parker et al., 2020; Wimberger et al., 2017). The dependence of samango monkeys on this habitat type therefore explains the dominant selection toward this landscape variable over other variables across all orders of selection. Such a strong selection for areas including important fruit species when feeding has important conservation implications, as many primate species have been observed to decline significantly when key food species are removed through logging or other anthropogenic processes (Chapman et al., 2006).

Avoidance of human settlements was also a significant predictor when selecting ranging areas. Avoidance of areas of increased human use can predominantly be attributed to the lack of forest cover associated with these areas, a requirement for arboreal species, but has also been widely observed in other animals (ungulates: Theuerkauf & Rouys, 2008; cougars: Knopff et al., 2014; leopards: Pitman et al., 2017; macaques: Waterman et al., 2019). Although samangos are capable of utilizing exotic species in residential gardens (Nowak et al., 2017; Wimberger et al., 2017) and consuming human waste (Linden et al., 2016), these areas are inherently “riskier” due to the lack of cover provided from natural predators and the risk of conflict with humans (Lawes, 1991; Lawes et al., 1990; Nowak et al., 2014, 2017). Furthermore, areas closer to human settlements increase the risk of mortality from road collisions (Linden et al., 2020), while hunting for bushmeat and traditional medicine also becomes more prevalent (Linden et al., 2016; Sawyer & Brashares, 2013). Human settlements did not influence probability of use within the home range, in contrast to other studies (Fattebert et al., 2015; Sawyer & Brashares, 2013), possibly as a result of exploring use at this finer scale of interest. However, the area from which the available sample was drawn at this scale may also have influenced coefficient estimates compared with other studies, which sampled availability from a broader extent (Fattebert et al., 2015; Sawyer & Brashares, 2013).

Despite the inherent risk associated with areas of increased human activity (Nowak et al., 2017), samango monkeys positively selected for these areas when feeding. However, selection for human settlements at this scale may simply be an artifact of sampling availability from a broader extent compared with 3rd‐order selection, particularly the influence of some “available” locations at this order on the north‐facing side of the mountains further from human settlements, compared with “used” locations on the south‐facing side of the mountains closer to settlements. However, as the nearest human settlement to the study groups is located at the base of the mountain range (Figure 2), and is therefore inaccessible, the positive selection observed here is likely to be a consequence of the location of our groups to the proximity of the nearest human settlement. Despite this, human settlements may be important for other samango monkey populations. Samango monkey groups in the Amathole Mountains in the Eastern Cape have been shown to utilize exotic fruits in residential gardens (Wimberger et al., 2017), particularly when the availability of natural resources within forest fragments is depleted. Furthermore, human settlements provide monkeys with additional feeding opportunities through food waste (Linden et al., 2016; Wimberger et al., 2017), increasing the potential of monkeys to utilize human‐dominated areas when natural resources are scarce.

Terrain ruggedness was a significant predictor of samango monkey use across all scales of selection. Samango monkeys preferred areas of higher terrain ruggedness when establishing home ranges, likely a consequence of avoiding areas off the mountain range, which are more open and associated with increased human activity (Mostert et al., 2008). In contrast, areas of high terrain ruggedness were avoided at finer scales of selection. Within the home range, samangos are reluctant to climb steep, open cliff faces as their core ranging area tends to be associated with the indigenous forest at the base of these cliffs. Ranging intensity also declines as they move downslope from these areas (Parker et al., 2020). Furthermore, these more rugged areas lack the dense vegetation associated with preferred indigenous fruit species (Linden et al., 2015; Nowak et al., 2017; Parker et al., 2020; Wimberger et al., 2017) and are inherently “riskier” (Coleman & Hill, 2014b; Parker et al., in review).

Distance to main rivers influenced the probability of use at the landscape level, with samangos preferring to establish ranges closer to rivers. Riverine habitats are known to be important to various species (Pitman et al., 2017), including samangos, due to their vegetative structure and composition (Lawes, 1992; Linden et al., 2016; Skinner & Chimimba, 2005), which includes important fruit species in the samango monkey diet (Linden et al., 2015). Distance to rivers dropped out our RSFs at finer scales of selection due to the collinearity with elevation, which was more strongly selected for. The use within the home range and when feeding was positively associated with higher elevations, likely an indirect result of the preference toward the tall‐canopy indigenous forest, which, even within the home range, occurred at higher elevations along the base of the south‐facing cliffs of the mountain (Mostert et al., 2008).

Projection of the scale‐integrated RSF (SRSF) across Limpopo identified three key conservation areas for samango monkeys based on probability of use and current distribution records of samango monkey populations across Limpopo (Dalton et al., 2015; Lawes, 1990, 1992; Linden et al., 2016). Using a probability of use threshold above 2/3 to delineate critical habitat (Heinrichs et al., 2010), we found that the area of critical habitat across Limpopo was 431.2 km^2^, an estimate similar to the area of occupancy for *C. a*. *schwarzi* given in Linden et al. (2016). The Woodbush Forest Reserve (Figure 3b) comprised the largest area of critical habitat across Limpopo and was also the conservation area with the greatest mean patch area, confirming its importance for samango monkey conservation (Linden et al., 2016).

The Mariepskop area (Figure 3c), which represents the southern range limit of *C. a*. *schwarzi* in Limpopo, was the key conservation area with the smallest area of critical habitat. However, there is potential for Mariepskop subpopulations to connect to those in the Woodbush area through possible suitable habitat along the escarpment between these subpopulations. Furthermore, suitable habitat along the escarpment to the south of Mariepskop may also connect these subpopulations to those in Swaziland.

Despite the Soutpansberg Mountains covering the largest extent of all the conservation areas (Figure 3a), critical habitat was less than that of the Woodbush Forest Reserve, comprising just 98.1 km^2^. The Soutpansberg Mountains also had the greatest number of individual patches and smallest mean patch area, which was reflected in the projection of critical habitat across the mountain range. The SRSF also delineated that the majority of suitable habitat was located toward the east of the mountain range, between the towns of Louis Trichardt and Thohoyandou where patches are larger and more contiguous than those in the west. These forest patches are therefore particularly crucial to the long‐term persistence of samangos in the Soutpansberg, yet are under greater pressure from human development (Linden et al., 2016). In the western Soutpansberg, however, pronounced fragmentation of critical habitat may further necessitate the need for samango monkeys to utilize matrix habitat between forest fragments (Parker et al., 2020).

It is important to note that the coarser resolution in which probability of use was projected across Limpopo (250 m^2^/0.06 km^2^), compared with the Soutpansberg Mountains (30 m^2^/0.0009 km^2^), would have impacted our projection and likely resulted in larger, more fragmented patches of critical habitat. However, projecting critical habitat at this coarser scale is more representative of the minimum critical forest patch size required for samango monkey persistence (0.44 km^2^) (Lawes et al., 2000). This is particularly evident when considering patches of connecting suitable matrix habitat, which, as these patches were below our critical habitat threshold, were not included in our projections.

Projection of the SRSF across Limpopo indicates that the Soutpansberg subpopulations of *C. a*. *schwarzi* are isolated from the escarpment subpopulations due to lack of suitable connecting habitat and anthropogenic landscape change. Based on our projection of suitable habitat across Limpopo, it is increasingly likely that complete separation exists between northern *C. a*. *schwarzi* subpopulations in the Soutpansberg and southern *C. a*. *schwarzi* subpopulations in the Woodbush and Mariepskop areas, as suggested by Linden et al. (2016). However, our projection also identifies a fourth potential conservation area between the towns of Elim and Morebeng (Figure 3d). While this area appears to contain large patches of suitable habitat, there are no existing records of samango monkeys at this location to our knowledge. In the absence of local samango subpopulations, the area could still provide opportunities for connectivity between the Soutpansberg and Woodbush subpopulations, or perhaps serve as a location for reintroductions to establish additional subpopulations following suitable habitat assessments.

An important consideration of this study is that resource selection was modeled based on location data of two samango monkey groups, which places limitations on the projections. Ranging data from other populations, particularly those in the eastern part of the mountain range near Thohoyandou and in the Woodbush Forest Reserve, could significantly improve resolution. These results would also benefit from the integration of location data from other samango subspecies, such as *C. a*. *erythrarchus* in KwaZulu Natal and *C. a*. *labiatus* in the Eastern Cape (Dalton et al., 2015), in order to prioritize samango habitat and inform management plans across South Africa. Integration of data from other groups may also highlight potential differences in resource selection between conspecifics (Morato et al., 2018), although we would still expect primary productivity to consistently be the landscape feature most strongly selected for across all orders of selection, owing to the dependence of samangos on tall‐canopy indigenous forests. Indeed, analysis for other species has shown consistent patterns of resource selection across Limpopo Province (Pitman et al., 2017). Finally, conservation efforts would greatly benefit from confirmation of presence/absence of samango populations in the area identified around Morebeng, in addition to the development of connectivity models and genetic analysis among subpopulations, to ensure the long‐term viability of this endangered species through the protection of suitable habitat that links populations.

## 5 CONCLUSION

In conclusion, the results from our study show that samango monkey distribution across Limpopo is highly fragmented and ultimately limited by the availability of suitable habitat. Our SRSF confirms three key conservation areas for samango monkeys in Limpopo, while also outlining the potential separation of northern subpopulations of *C. a*. *schwarzi* in the Soutpansberg from southern populations in Woodbush and Mariepskop due to the lack of suitable connecting habitat. Ongoing deforestation in indigenous forest regions and riverine habitats for commercial timber operations is therefore the greatest, most immediate threat facing samango monkeys across South Africa (Lawes, 2002; Lawes et al., 2000; Linden et al., 2016). As the global human population continues to grow, further fragmentation from increased urbanization and corresponding road networks also represents a significant threat to this species (Linden et al., 2016, 2020). Further to presenting significant barriers to dispersal, road networks also result in frequent fatalities of individuals moving between forest fragments (Linden et al., 2020). This fragmentation presents a major threat to a species which is unable to recolonize forest patches and is susceptible to local extinctions in small forest fragments (Lawes, 2002; Lawes et al., 2000), due to the poor dispersal capabilities of samangos and their reluctance to travel over open ground (Lawes et al., 2000). This is of particular concern to *C. a*. *schwarzi,* which, of the three samango monkey subspecies, typically occupy the largest home ranges (Linden et al., 2016).

## CONFLICT OF INTEREST

The authors declare no conflict of interest.

## AUTHOR CONTRIBUTIONS

**Edwin J. Parker:** Conceptualization (equal); Data curation (lead); Formal analysis (lead); Investigation (lead); Methodology (equal); Visualization (equal); Writing‐original draft (lead). **Nicola F. Koyama:** Conceptualization (equal); Formal analysis (equal); Funding acquisition (equal); Methodology (equal); Project administration (equal); Resources (equal); Supervision (equal); Visualization (equal); Writing‐original draft (equal). **Russell A. Hill:** Conceptualization (equal); Formal analysis (equal); Funding acquisition (equal); Methodology (equal); Project administration (equal); Resources (equal); Supervision (equal); Visualization (equal); Writing‐original draft (equal).

## Data Availability

Data are available from the Dryad Digital Repository at https://doi.org/10.5061/dryad.dr7sqv9xw.
